# County-Level Variation in Triple Guideline-Directed Medical Therapy in Heart Failure With Reduced Ejection Fraction

**DOI:** 10.1016/j.jacadv.2024.101014

**Published:** 2024-06-12

**Authors:** Rishi J. Desai, Danielle Stonely, Naira Ikram, Raisa Levin, Ankeet S. Bhatt, Muthiah Vaduganathan

**Affiliations:** a Division of Pharmacoepidemiology and Pharmacoeconomics, Department of Medicine, Brigham and Women’s Hospital, Harvard Medical School, Boston, Massachusetts, USA; b Department of Cardiology, Kaiser Permanente San Francisco Medical Center, San Francisco, California, USA; c Division of Cardiovascular Medicine, Department of Medicine, Brigham and Women’s Hospital, Harvard Medical School, Boston, Massachusetts, USA

**Keywords:** geographic variation, guideline-directed medical therapy, heart failure, reduced ejection fraction

## Abstract

**Background:**

Current guidelines recommend simultaneous initiation of multidrug guideline-directed medical therapy classes for heart failure with reduced ejection fraction.

**Objectives:**

The purpose of this study was to evaluate county-level variation in use of triple guideline-directed medical therapy, defined as simultaneous prescription fills for beta-blockers, renin-angiotensin system inhibitors or angiotensin receptor neprilysin inhibitors, and mineralocorticoid receptor antagonists, in heart failure with reduced ejection fraction.

**Methods:**

We conducted a cohort study using Medicare Fee-for-Service claims data (parts A, B, and D between 2013 and 2019). Features of counties including area-level indicators of poverty, employment, and educational attainment and aggregated patient-level sociodemographic and medical history variables were compared by quintiles of triple therapy use. A multilevel logistic regression model was constructed to estimate the contextual effect of clustering by counties, which was expressed as a median OR.

**Results:**

304,857 patients from 2,600 counties (83% of all U.S. counties) were included. The median for triple therapy use was 14.3% (IQR: 10.3%-18.8%) across included counties with a wide variation (range: 0%-54.5%). Compared to counties in the highest use quintile, counties in lowest triple therapy use quintile had worse area-level indicators of socioeconomic status (% unemployment 6.8% vs 6.2%). Counties in lowest quintile had higher proportion of Black patients (13.3% vs 5.7% in highest quintile) and patients with low-income subsidy (29.3% vs 25.8% in highest quintile). The median OR was 1.30 (95% CI: 1.28-1.33).

**Conclusions:**

We observed variation in triple therapy use across counties in the United States with suboptimal local use patterns correlating with indicators of socioeconomic disadvantage.

In patients with heart failure with reduced ejection fraction (HFrEF), consensus guidelines recommend rapid sequence initiation of available guideline-directed medical therapy (GDMT), including evidence-based beta-blockers, renin-angiotensin system inhibitors (RASi) or angiotensin receptor neprilysin inhibitors, mineralocorticoid receptor antagonists (MRAs), and sodium-glucose co-transporter 2 inhibitors (SGLT2).^1^ Prior research has documented substantial variation in use of GDMT among patients with HFrEF,^2^^,^^3^ yet there is a paucity of information regarding the use of multidrug GDMT regimen in nationally representative cohorts of patients with HFrEF treated in routine clinical care.

Geographic variation in outcomes of HF including reduced and preserved ejection fraction subtypes, including HF-related mortality, is well described;^4^^,^^5^ yet, little is known regarding geographic variation in treatment utilization in the United States. One study based on the Get With The Guidelines–Heart Failure (GWTG-HF) registry noted no differences in quality of care measures, which included GDMT use, across census regions in the United States.^6^ However, grouping of geographic regions into just 4 broad census regions may have masked important intra-region differences. A more recent investigation conducted in patients from the Veteran’s Affairs revealed important geographic variation in GDMT use; however, owing to the peculiarities of the Veteran’s Affairs health system which includes ∼98% males and provides a generally superior access to health care services,^7^ it is unclear if those findings are generalizable to the U.S. population. Medicare enrollees represent a critically important population to investigate for HFrEF since a large majority of these patients are insured with this program.^8^ The burden of worsening HF in Medicare enrolled patients with HFrEF in routine clinical care is substantial with 1-year cumulative incidence estimated to be 42.3%.^9^ It is plausible to hypothesize that with optimization of GDMT in this population, a portion of this high burden may be addressable. In this study, we aimed to address this knowledge gap and describe utilization of GDMT classes at county level in a population-based cohort of Medicare enrollees across the United States using data from 2013 to 2019. Since SGLT2 inhibitors were only recently approved for the indication of HFrEF, we restricted this evaluation to use of triple therapy consisting of beta-blockers, RASi (including angiotensin receptor blockers, angiotensin-converting enzyme inhibitors) or sacubitril/valsartan, and MRA.

## Methods

### Data source and study cohort

Medicare fee-for-service claims data (2013-2019) from part A (inpatient coverage), part B (outpatient coverage), and part D (prescription medications) were used to conduct this study. A cohort of patients including incident and prevalent HF cases was assembled based on diagnosis codes of HF in inpatient or outpatient claims. Cohort entry date was defined as date of a medical encounter with a recorded HF diagnosis after a 6-month baseline period of continuous enrollment in Medicare parts A, B, and D. We required patients to have procedure codes for echocardiography or cardiac catheterization, in the 30 days prior to (and including) the cohort entry date, to improve the specificity of HF diagnosis and ensure close contact with the health care system.^9^ Given the lack of EF results availability, we then applied a validated claims-based probabilistic phenotyping model using 35 predictors identified in the baseline period to classify patients into HFrEF vs heart failure with preserved ejection fraction (HFpEF) (overall accuracy 82%; positive predicted value for HFrEF 73% in internal and external validation).^10^^,^^11^ This study was approved by the Brigham and Women’s Hospital Institutional Review Board (protocol #2019P001953).

### Outcome assessment

We assessed the use of triple GDMT, defined as dispensed prescriptions of 3 GDMT classes, beta-blockers, RASi (including angiotensin receptor blockers, angiotensin-converting enzyme inhibitors) or sacubitril/valsartan, and MRA, as the outcome of interest in the study cohort within 90 days of cohort entry with censoring on disenrollment from Medicare part A, B, or D, or mortality.

### County-level aggregation

Patients were assigned to counties based on their residential Zone Improvement Plan codes identified from enrollment files. We excluded counties with <10 patients as a variance control measure. County level use of triple therapy was determined based on percentage of included patients within each county on triple therapy.

### Study variables

We defined the following county-level variables to evaluate features of counties with varying use of GDMT: 1) socioeconomic status (SES) indicators: percentage unemployed, percentage below the poverty line, median value of owner-occupied homes, median household income, and percentage with <12th grade education (all derived from American Community Survey by zip code);^12^ county level summaries were derived based on averaging across zip codes within counties; 2) geographic census region; and 3) rurality of counties based on rural-urban continuum coding.^13^

Additionally, we aggregated key patient-level sociodemographic (age, race, gender, low-income subsidy recipient status) and medical history variables identified from Medicare claims within each county to describe the county level case-mix. Medical history variables included heart failure hospitalizations, atrial fibrillation, chronic obstructive pulmonary disease, dementia, diabetes, hyperkalemia, hypotension, myocardial infarction, and renal dysfunction and were assessed during the 6-month baseline period for each patient based on International Classification of Diseases codes recorded on medical claims.

### Statistical analysis

To describe the use of GDMT at the county level, quintiles were created based on percentage of patients on triple HF therapy within each county. County features and patient case-mix were described by triple therapy use quintiles using means and proportions averaged over counties. Use of triple therapy was also plotted on the U.S. map by counties to visually evaluate geographic variation.

To estimate the contextual effect of clustering by counties in use of GDMT, we fitted a multilevel logistic regression model with triple therapy use as the outcome variable including random effects for counties and fixed effects for patient medical history variables described above and demographics (age, gender) as independent variables. We did not include potential patient-level indicators of disparities (race, low-income subsidy status) as well as county-level features summarizing aggregate measures of SES and rurality in the multilevel logistic model as adjustment on such factors implies an intervention and potentially distorted reality.^14^ For instance, if we include indicators such as median household income in the adjustment set, it estimates levels of GDMT use at county level in a hypothetical world where the household incomes are held constant across counties. Therefore, such adjustment could understate the magnitude of differences in use of GDMT owing to existing disparities. Model fit was evaluated using −2 log likelihood, and nested models were compared using the likelihood ratio test. Based on the estimate of between county variance from the model, we described the effect of clustering by counties on triple therapy use with 2 measures: 1) variance partition coefficient calculated based on the latent response formulation,^15^^,^^16^ as a representation of the proportion of the total observed variation in triple therapy use attributable to between-cluster variation; and 2) median OR (MOR) to quantify the magnitude of the effect of clustering by counties.^15^ MOR can be interpreted as median increase in odds of triple therapy receipt if a patient moved from a randomly selected county with lower prevalence of triple therapy use to a county with higher prevalence. MOR values range from 1.0 to infinity and values close to 1.0 indicate low heterogeneity between geographic areas, while higher values indicate more heterogeneity.

## Results

### Study sample

A total of 315,140 patients met all our inclusion criteria from 3,135 counties (out of total 3,143 counties in the United States). Excluding counties with <10 patients or missing county-level SES data resulted in restriction to 304,857 patients from 2,600 counties, which comprised the final study sample. The median number of patients per county was 45 (IQR: 24-101).

### Triple therapy use

**Figure 1:**
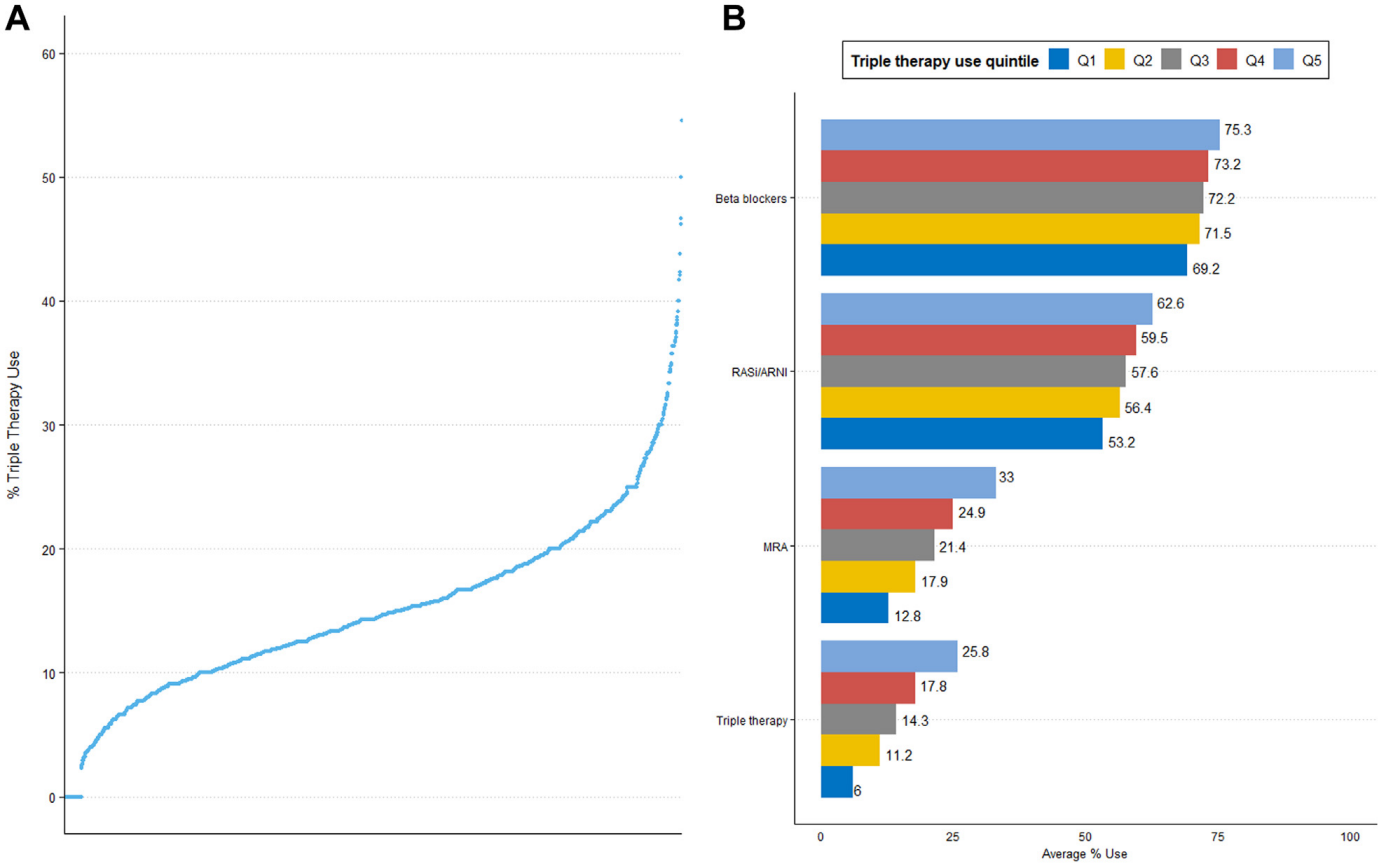
**Guideline-Directed Medical Therapy Use in Heart Failure With Reduced Ejection Fraction, Medicare Data 2013 to 2019** (A) Variation in % of patients on triple therapy across counties, each dot represents a county (n = 2,600) (B) Use of guideline-directed medical therapy by counties in quintiles of therapy use. ARNI = angiotensin receptor neprilysin inhibitor; MRA = mineralocorticoid receptor antagonist; RASi = renin-angiotensin system inhibitor.

**Figure 2:**
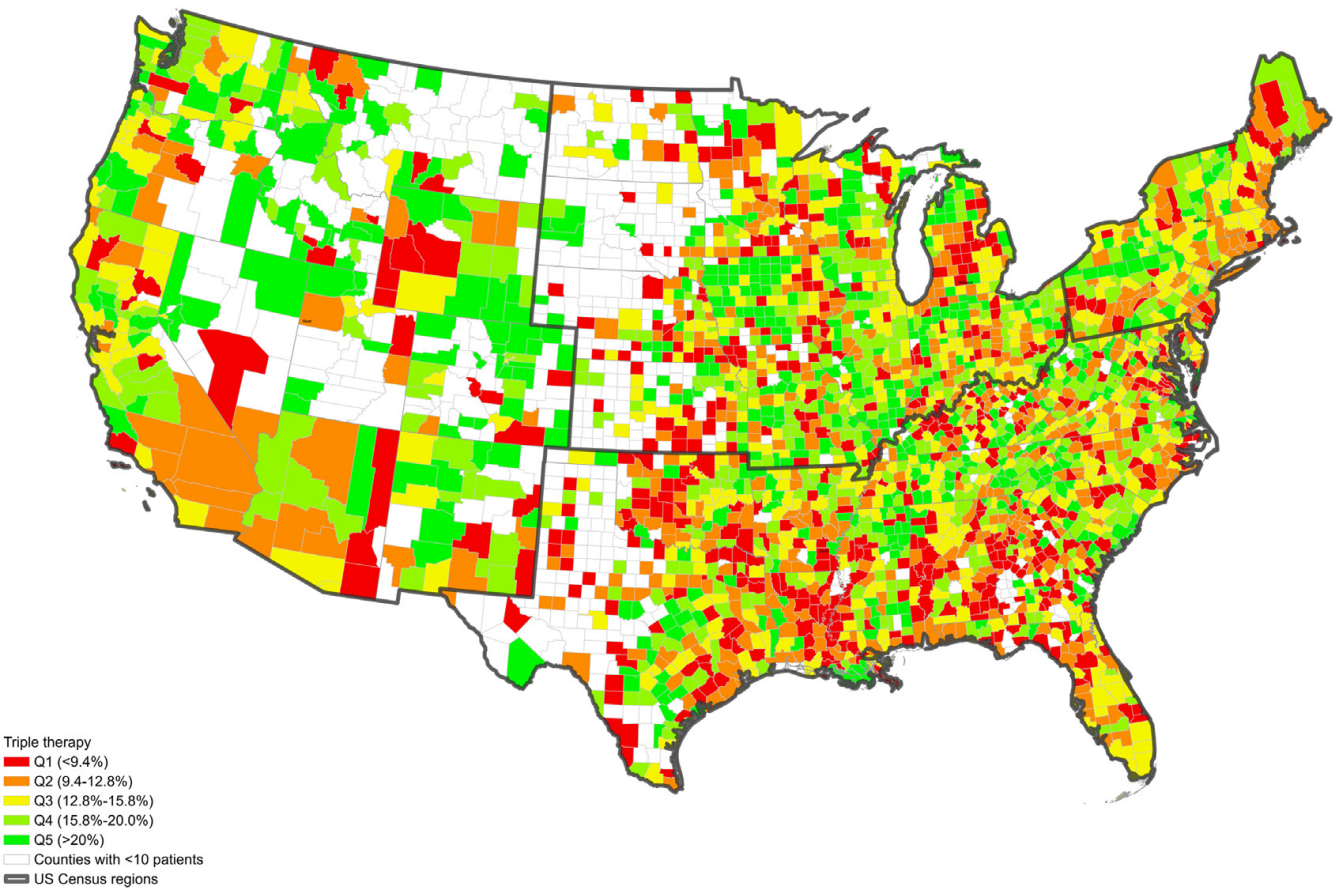
**Triple Therapy Use by Counties in Contiguous United States, Medicare Data 2013 to 2019** Among 1,262 included counties in the south, 25.4% were in the lowest use quintile (Q1, triple therapy use <9.4%); while relatively lower proportions were in q1 in other regions (11.6% of 216 in the northeast, 12.0% of 292 in the west, and 17.6% of 830 in the midwest).

Among the final sample of 304,857 patients, 43,108 (14.1%) were identified as triple therapy users. Across included counties, the median (IQR) for triple therapy use was 14.3% (10.3%-18.8%). A wide variation in patients on triple therapy was observed across included counties ranging from 0% to 54.5% (Figure 1A). Among the individual GDMT classes, beta-blockers were used most frequently and MRA least frequently (Figure 1B). Counties in the lowest quintile of triple therapy appeared to be concentrated in the South (Figure 2).

### County-level characteristics by triple therapy use quintiles

Counties in lowest triple therapy use quintile had worse area-level indicators of poverty, employment, and educational attainment compared to counties in higher use quintiles (Table 1, Central Illustration). A higher proportion of the counties in the lowest quintile were classified as completely rural or urban areas with <2,500 population based on rural-urban continuum coding.

**Table 1:** County-Level Characteristics by Triple Therapy Use Quintiles

	Q1 (Lowest)	Q2	Q3	Q4	Q5 (Highest)
Total counties	518	522	520	530	510
Triple therapy use range	<9.4%	9.4-12.8%	12.8-15.8%	15.8-20.0%	>20.0%
County features					
Socioeconomic status indicators					
Average % below poverty line	17.5%	15.8%	15.4%	16.2%	15.7%
Average % unemployment	6.8%	6.6%	6.5%	6.6%	6.2%
Average % with <12th grade education	36.1%	33.9%	33.1%	34.3%	35.2%
Average median household income rank (1 [lowest] to 100 [highest])	37	45	46	43	41
Average median value of homes rank (1 [lowest] to 100 [highest])	38	46	48	45	43
Rural-urban continuum coding					
% counties with completely rural or urban population <2,500	16.2%	7.9%	7.5%	9.6%	13.1%
% counties with urban population 2,500-250,000	50.2%	39.1%	40.2%	46.4%	56.5%
% counties in metro areas of >250,000 population	33.6%	53.1%	52.3%	44.0%	30.4%
Patient-level case-mix					
Total number of patients	30,216	99,299	93,082	55,063	27,197
Sociodemographics					
Average age (y)	75	76	76	75	75
% male	67.5%	67.9%	67.4%	67.8%	67.5%
% Black race	13.3%	12.8%	12.6%	11.0%	5.7%
% White race	82.3%	79.6%	81.4%	84.1%	91.2%
% with low-income subsidy	29.3%	31.6%	28.0%	26.8%	25.8%
Medical history					
% with heart failure hospitalization	20.2%	20.8%	20.9%	19.8%	18.5%
% atrial fibrillation	52.7%	53.2%	53.5%	53.0%	53.3%
% COPD	36.1%	33.6%	33.5%	33.5%	34.6%
% dementia	10.7%	11.6%	11.2%	10.0%	8.9%
% diabetes	49.0%	49.3%	47.8%	46.9%	45.1%
% hyperkalemia	11.3%	11.7%	11.3%	10.8%	10.2%
% hypotension	20.2%	19.3%	19.8%	19.7%	19.7%
% myocardial infarction	31.6%	31.3%	31.4%	30.7%	31.2%
% renal dysfunction	26.0%	26.9%	26.5%	25.1%	22.5%

COPD = chronic obstructive pulmonary disease.

**Central Illustration:**
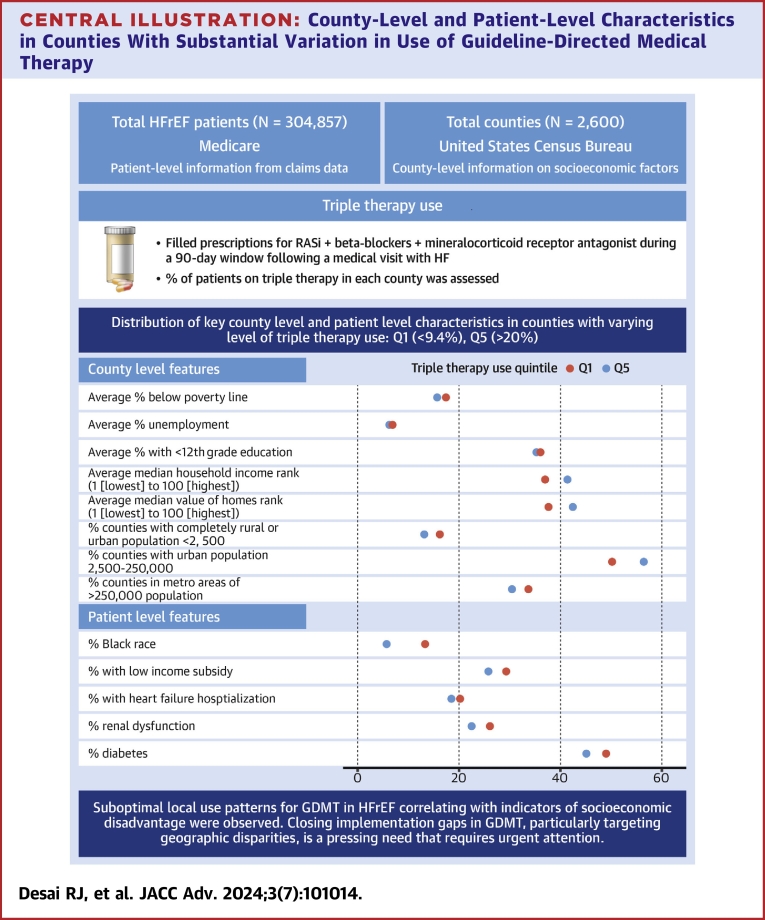
**County-Level and Patient-Level Characteristics in Counties With Substantial Variation in Use of Guideline-Directed Medical Therapy** GDMT = guideline-directed medical therapy; HFrEF = heart failure with reduced ejection fraction; RASi = renin-angiotensin system inhibitors.

Among patient-level sociodemographic factors, proportion of Black patients and patients with low-income subsidy was higher in lower use quintiles. Among medical history variables, counties in the lowest use quintile tended to have a higher burden of comorbid conditions including diabetes, renal dysfunction, and chronic obstructive pulmonary disease.

### Contextual effect of clustering by counties in use of triple therapy

In the multilevel logistic regression model, we observed that model fit substantially improved after including random effects for counties compared to a fixed effects-only model (*P* value for likelihood ratio test <0.0001). Based on the calculated variance partition coefficient, we noted that 2.3% (95% CI: 1.9%-2.6%) of the residual variation in triple therapy use that persisted after adjustment for included demographics and medical history variables was explained by systematic differences between counties. The MOR was 1.30 (95% CI: 1.28-1.33), suggesting that when comparing 2 identical patients from randomly selected counties, the odds of triple therapy use were 30% higher if the patient moved to higher use vs lower use county.

Table 2 provides OR and 95% CIs for the fixed effect variables which generally indicated lower odds of treatment with triple therapy among patients with various comorbid conditions. The effect sizes were especially large for history of conditions that indicate higher potential for adverse event risks with triple therapy including hyperkalemia (0.62 [95% CI: 0.60-0.65]), hypotension (0.76 [95% CI: 0.74-0.78]), dementia (0.64 [95% CI: 0.61-0.67]), and renal dysfunction (0.65 [95% CI: 0.63-0.67]).

**Table 2:** Patient-Level Characteristics and Their Association With Receipt of Triple Therapy

	Counts (%)	Multivariable OR (95% CI)^a^
Total sample	304,857 (100%)	
Age, y	76 ± 8	0.96 (0.96-0.97)
Male	206,250 (67.6%)	0.94 (0.92-0.97)
History of HF hospitalization	62,135 (20.4%)	1.15 (1.13-1.16)
Key comorbid conditions		
Chronic obstructive pulmonary disease	103,355 (33.9%)	0.90 (0.88-0.92)
Myocardial infarction	95,166 (31.2%)	0.88 (0.86-0.90)
Atrial fibrillation	162,186 (53.2%)	0.96 (0.94-0.98)
Diabetes	146,335 (48.0%)	0.96 (0.94-0.98)
Renal dysfunction	79,211 (26.0%)	0.65 (0.63-0.67)
Hypotension	59,938 (19.7%)	0.76 (0.74-0.78)
Hyperkalemia	34,276 (11.2%)	0.62 (0.60-0.65)
Dementia	33,088 (10.8%)	0.64 (0.61-0.67)

Values are n (%) or mean ± SD unless otherwise indicated.

a Estimates from the multilevel logistic regression model with triple therapy use as the outcome variable including random effects for counties and fixed effects for variables in this table.

## Discussion

In a nationally representative sample of Medicare enrollees including patients from 2,600 counties (83% of the total counties in the United States), we observed that fewer than 1 in 7 patients with HFrEF were on triple therapy. We also documented appreciable geographic variation across counties and identified several features of counties with low observed use including worse indicators of area-level SES, higher density of Black patients, and greater medical complexity.

Geographic variation in mortality related to heart failure, including reduced and preserved ejection fraction subtypes, in the United States is well-described.^4^^,^^5^ A state-level analysis found that Southern states of Alabama, Mississippi, Oklahoma, and Arkansas consistently had among the highest age-adjusted mortality rates between 1999 and 2017.^4^ Our findings suggest that some of these same geographic regions also lag in implementation of optimal GDMT, which represents an important opportunity to address the variation in mortality observed in prior studies. As clinical evidence and practice guidelines strongly support multidrug, comprehensive medical therapy initiated simultaneously or in rapid sequence, it is critical to consider and address existing disparities. Importantly, our analysis demonstrates that the most vulnerable and high-risk patients, including those in communities with the greatest socioeconomic disadvantages and those with multimorbid cardiometabolic conditions, continue to be those least well served in terms of implementation of disease-modifying HF therapy.^17^^,^^18^ While a complex set of issues related to access, health literacy, and affordability may play a role in explaining our observations, it is important to explicitly consider the known disparities when developing and evaluating novel interventions to bridge implementation gaps in care of HF patients.^19^^,^^20^ In counties with suboptimal use, community-based interventions, such as those which have proven effective in other disease states,^21^ hold promise and require prospective evaluation.

Our findings documenting low use of GDMT in HFrEF add to an existing body of literature detailing pervasive implementation gaps in the United States. In an U.S.-based ambulatory HF registry CHAMP (Change the Management of Patients with Heart Failure), Greene et al^2^ previously reported that fewer than 1 in 4 patients were treated simultaneously with beta-blockers, RASi, and MRAs. Recent multinational data found these implementation gaps have not been closed in more contemporary time periods and similarly exists in other countries.^22^ Another study identified that among Veterans with HFrEF, racial and ethnic minority patients were not less likely to receive GDMT; however, they were less likely to be treated with appropriate dose uptitration to target.^3^

An important consideration when evaluating real-world use patterns of medications is cost implications for patients. Modeling studies have demonstrated multidrug regimen to be cost-effective at $150,000 per quality adjusted life year threshold in HFrEF.^23^^,^^24^ However, the cost burden on patients remains high even after Medicare coverage, especially for treatments that are not available as generics including sacubitril/valsartan and SGLT2 inhibitors with an average out of pocket costs estimated to be about $1,300 annually for triple therapy and $2,200 for quadruple therapy when these 2 medications are used as parts of the multidrug regimen.^25^ While the low use of triple therapy we observed in our study could be partly explained by high cost sharing for sacubitril/valsartan, we note that use of other RASi options including angiotensin converting enzyme inhibitors and angiotensin receptor blockers which are available as generics has relatively low out-of-pocket burden with an estimated annual cost of $159 to the patients with Medicare coverage.^25^ Therefore, our findings are likely not fully explained by financial deterrence. Some degree of clinical inertia, lack of knowledge or urgency among providers, and reservations due to increased side effect burden may play a role in explaining low triple therapy use observed in our study. Furthermore, in 2025 under the provisions of inflation reduction act, out-of-pocket cost will be capped at $2,000 annually for Medicare beneficiaries, which should help avoiding cost-related nonuse of medication to some extent.^26^

Some strengths and limitations of the current study deserve discussion. Representativeness, including a national sample of patients with HFrEF treated in ambulatory care settings and hospitals is a major strength of the current study; to our knowledge, this is the largest study assessing county-level variation in the uptake of GDMT in patients with HF. Reliable capture of medication use through prescription dispensing records is another strength. However, misclassification of HF phenotype and comorbid conditions is possible in the context of this claims-based analysis. For distinguishing between HFrEF and HFpEF, we relied on a probabilistic phenotyping algorithm which could have misclassified some HFpEF patients into our cohort. As HFpEF patients are not likely to be initiated on triple therapy due to lack of evidence of effectiveness, this misclassification could result in underestimation of triple therapy use in our cohort. The time period of our study predated the evidence supporting SGLT2i in HF, and therefore uptake of this class of medications was not included in this analysis. Variation in SES and other factors may exist within counties at the local/municipal level, which was not captured in this analysis. We also excluded counties with <10 patients, which may have led to exclusion of smaller or less densely population counties where disparities may be greater. We were restricted to information on medication dispensing available through pharmacy claims, which do not capture dispensing records where patient fully paid for the medications out of pocket. Another limitation is that we did not exclude special populations such as end-stage renal disease who may be not treated with triple therapy classes due to tolerability concerns. Finally, our observations may not generalize to non-Medicare patient populations. Specifically, due to use of Medicare data, our cohort included patients with HFrEF with an average age of 76 years. Among older patients, treatment decisions are often influenced by considerations related to adverse events of medications. In our multivariable models, we observed history of hyperkalemia, renal dysfunction, and hypotension to be associated with lower odds of triple therapy use. Therefore, in younger populations who may have lower baseline risk of adverse events, triple therapy use may be higher.

## Conclusions

Combination use of 3 GDMT classes was infrequent in patients with HFrEF and subject to marked variation across counties correlating with indicators of socioeconomic disadvantage. Closing implementation gaps in GDMT, particularly targeting geographic disparities, is a pressing need that requires urgent attention.

PERSPECTIVES **COMPETENCY IN PATIENT CARE:** In patients with HFrEF, consensus guidelines recommend rapid sequence initiation of available GDMT.

**TRANSLATIONAL OUTLOOK:** In a nationally representative sample of Medicare enrollees including patients from 2,600 counties (83% of the total counties in the U.S.), we observed that fewer than 1 in 7 patients with HFrEF were on 3 GDMT classes concurrently. We also documented appreciable geographic variation across counties and identified several features of counties with low observed use including worse indicators of area-level SES, higher density of Black patients, and greater medical complexity. Effective implementation of GDMT will require addressing structural and environmental barriers to access.

## Funding support and author disclosures

Dr Desai has served as principal investigator on research grants to Brigham and Women’s Hospital from Vertex, 10.13039/100008272Novartis, and 10.13039/100015340Bayer. Dr Vaduganathan has received research grant support or served on advisory boards for 10.13039/100016473American Regent, 10.13039/100002429Amgen, AstraZeneca, Bayer AG, Baxter Healthcare, 10.13039/100001003Boehringer Ingelheim, 10.13039/100014941Cytokinetics, Lexicon Pharmaceuticals, 10.13039/100008272Novartis, Pharmacosmos, 10.13039/501100015002Relypsa, 10.13039/100016545Roche Diagnostics, 10.13039/100004339Sanofi, and Tricog Health; has speaker engagements with AstraZeneca, 10.13039/100008272Novartis, and 10.13039/100016545Roche Diagnostics; and participates on clinical trial committees for studies sponsored by Galmed, 10.13039/100008272Novartis, Bayer AG, Occlutech, and 10.13039/100019443Impulse Dynamics. All other authors have reported that they have no relationships relevant to the contents of this paper to disclose.
